# Effect of Stretching of Spastic Elbow Under Intelligent Control in Chronic Stroke Survivors—A Pilot Study

**DOI:** 10.3389/fneur.2021.742260

**Published:** 2021-12-14

**Authors:** Sanjana Rao, Meizhen Huang, Sun Gun Chung, Li-Qun Zhang

**Affiliations:** ^1^Department of Physical Therapy and Rehabilitation Science, School of Medicine, University of Maryland, Baltimore, MD, United States; ^2^Department of Rehabilitation Medicine, Seoul National University, Seoul, South Korea; ^3^Department of Orthopaedics, School of Medicine, University of Maryland, Baltimore, MD, United States; ^4^Department of Bioengineering, University of Maryland, College Park, MD, United States

**Keywords:** spasticity, stroke, rehabilitation, elbow, tendon reflex

## Abstract

**Objective:** To assess the short-term effects of strenuous dynamic stretching of the elbow joint using an intelligent stretching device in chronic spastic stroke survivors.

**Methods:** The intelligent stretching device was utilized to provide a single session of intensive stretching to the spastic elbow joint in the sagittal plane (i.e., elbow flexion and extension). The stretching was provided to the extreme range, safely, with control of the stretching velocity and torque to increase the joint range of motion (ROM) and reduce spasticity and joint stiffness. Eight chronic stroke survivors (age: 52.6 ± 8.2 years, post-stroke duration: 9.5 ± 3.6 years) completed a single 40-min stretching intervention session. Elbow passive and active ROM, strength, passive stiffness (quantifying the non-reflex component of spasticity), and instrumented tendon reflex test of the biceps tendon (quantifying the reflex component of the spasticity) were measured before and after stretching.

**Results:** After stretching, there was a significant increase in passive ROM of elbow flexion (*p* = 0.021, *r* = 0.59) and extension (*p* = 0.026, *r* = 0.59). Also, elbow active ROM and the spastic elbow flexors showed a trend of increase in their strength.

**Conclusion:** The intelligent stretching had a short-term positive influence on the passive movement ROM. Hence, intelligent stretching can potentially be used to repeatedly and regularly stretch spastic elbow joints, which subsequently helps to reduce upper limb impairments post-stroke.

## Introduction

Stroke is one of the leading causes of long-term motor disability in adults, with ~795,000 people experiencing a new or recurrent episode of stroke every year in the United States (1). Spastic hemiplegia is a common motor impairment post-stroke. Spasticity and muscle weakness often occur together and contribute to the disordered motor control (2). Classically, spasticity has been defined as “a motor disorder characterized by a velocity-dependent increase in tonic stretch reflexes with exaggerated tendon jerks, resulting from hyperexcitability of the stretch reflex, as one component of the upper motor neuron syndrome” (3). Recently, to better reflect the underlying pathophysiology, the characterization of spasticity has been extended beyond the velocity dependence. Wu et al. extended velocity dependence to *position as well as velocity dependence* (4). Li et al. extended velocity dependence to velocity as well as muscle length-dependent increase in resistance (5). It results from hyperexcitable descending excitatory brainstem pathways and the resultant exaggerated stretch reflex responses. Other related motor impairments, including abnormal synergies, inappropriate muscle activation, and anomalous muscle coactivation, coexist with spasticity and share similar pathophysiological origins (5, 6).

Spasticity is a complex clinical symptom including reflex and non-reflex components, with both components contributing toward the increased resistance (7–13). Though the primary lesion attributing to spasticity lies within the central nervous system, the changes in connective tissue that ensues with immobilization further contributes toward an increase in spasticity (14). Muscle weakness or paresis leads to immobility. Immobility in turn can start a vicious cycle of changes including peripheral soft tissue changes that reduce tissue compliance, potentiation of reflex mechanisms, and spasticity. Eventually, these peripheral changes lead to muscle fibrosis, and decreased range of motion and function (15). Particularly, the increase in stiffness seen in the spastic limb cannot solely be attributed to the presence of hyperactive reflex. The increased resistance to stretch could be attributed to the passive stiffness arising from the connective tissues, tendons, ligaments, and passive muscle properties (9).

The prevalence of spasticity is known to increase as the time from stroke increases. About 17–42.6% of the chronic stroke survivors report the persistence of spasticity in their extremities, with the elbow joint known to be more commonly affected (79%) as compared to the other joints (16). Pronounced spasticity in the elbow disrupts the functional use of the upper limb, such as eating and grooming (17). Effective management of elbow spasticity is an important clinical need because of its frequent prevalence and proclivity to reduce functional outcome (18–21).

A large variety of physiotherapeutic and occupational therapeutic interventions have been described in the literature to manage spasticity (16). Passive stretching exercises are among the commonly prescribed techniques to alleviate spastic symptoms and/or joint stiffness (22–26). Previous studies have suggested that stretching of a spastic muscle in stroke survivors helps in maximizing the range of motion of the affected joint, decreasing musculotendinous stiffness due to passive torque reduction, and subsequently reducing spasticity of the affected limb (27). Passive muscle stretching has also been shown to activate Golgi tendon organs and inhibit the excitability of alpha motor neurons (27, 28). Some studies have investigated the effect of stretching on stretch reflexes elicited by tendon tap reflex. The reflexes elicited by tendon tap are affected by changes in muscle spindle sensitivity and reduce reflex amplitude (29, 30). This suggests that stretching might decrease the sensitivity of muscle spindles in response to rapid mechanical perturbation. However, passive stretching in clinical settings is usually performed manually by therapists, which is laborious and requires strenuous manipulation of the limbs of patients. Over the years, certain mechanically driven devices have been developed to help stretch the joints within restricted ranges, thereby reducing the burden of this labor-intensive process on the therapists. However, most of the existing devices such as the Continuous Passive Motion (CPM) machine are controlled based on the joint position and move at a constant velocity (31). These devices are commonly set to move the joints in their flexible part of the range of motion (ROM), and therefore do not usually stretch into the extreme positions where the spasticity/contracture is severe (32, 33). On the other hand, setting a CPM machine too aggressively may risk injuring the joint due to the lack of control of the resisting torque generated by the soft tissues.

To stretch the spastic elbow joints strongly yet safely at their extreme joint positions, we developed an “Intelligent” stretching device that dynamically stretches the joints with quantitative feedback control of the torque resistance as well as stretching velocity (34). In contrast to the constant speed of the CPM devices, the stretching velocity of this intelligent stretching device decreases as the resistance to movement increases. Based on the resistance torque produced during the movement, the stretching device constantly adjusts the stretching velocity. This ensures that the device provides a strong stretching in a safe yet effective manner. Through this intelligent stretching approach, the stretching provided can be adjusted according to the spasticity/contracture of the individual stroke survivor. This stretching device can be used not only to stretch the joint with spasticity and/or contracture but also to quantitatively evaluate stretch-induced changes in the biomechanical properties of the joint. The purpose of this study was to assess the immediate effects of strenuous dynamic stretching of the elbow joint performed with the help of an intelligent stretching device in chronic stroke survivors with a spastic elbow.

## Materials and Methods

### Participants

Eight chronic stroke survivors (age (mean ± SD): 52.6 ± 8.2 years; 5 men and 3 women) who had stroke for more than 1 year (duration (mean ± SD): 9.5 ± 3.9 years) were recruited. Participants with first focal unilateral lesion, ischemic or hemorrhagic, with a modified Ashworth score (elbow flexors) >0 and deep tendon reflex (biceps) >2 were included. Participants who presented with severe pain in the affected limb (7 or more out of 10 in the self-rating scale) and those with severe cardiovascular conditions were excluded. All participants gave informed consent before participating in the study, which was approved by the local Institutional Review Board. Table 1 provides the baseline characteristics of the participants enrolled in this study.

**Table 1:** Baseline characteristics of participants.

**Subject**	**Age**	**Gender**	**TSO**	**Affected**	**MAS**	**DTR**
	**(Years)**		**(Years)**	**side**	**(elbow flexors)**	
1	63.1	M	6.7	R	2	3
2	40.3	F	8.0	L	3	3
3	45.9	M	7.5	R	3	4
4	62.0	M	14.1	L	1	4
5	55.5	M	72	L	1+	2
6	58.1	F	4.4	L	3	4
7	46.6	M	13.7	L	3	3
8	50.0	F	14.4	R	3	3
Mean	52.6		9.5			3.2
SD	7.7		3.6			0.6

*TSO, time since onset; MAS, modified Ashworth scale; DTR, deep tendon reflex*.

### Experimental Procedure

**Figure 1:**
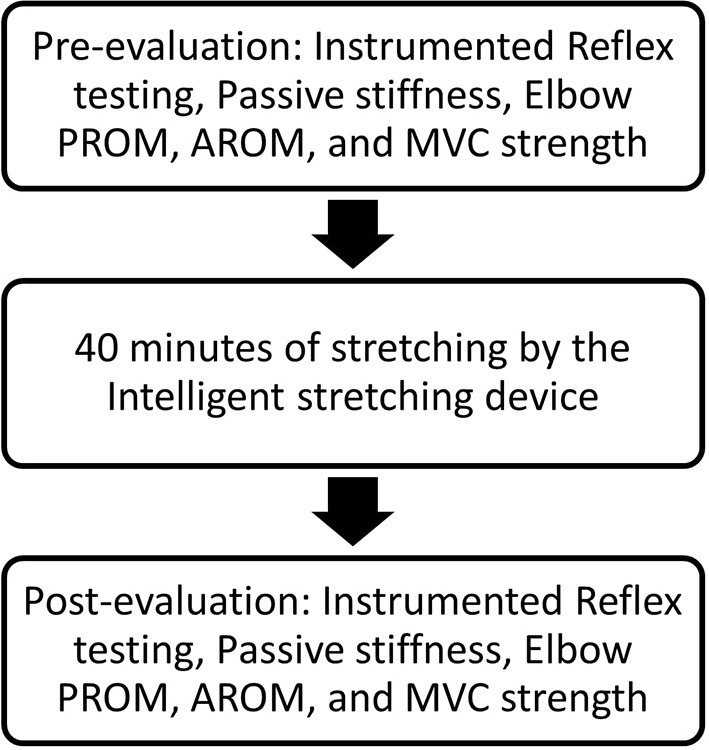
Representation of the flow of the experimental procedure. PROM, passive range of motion; AROM, active range of motion; MVC, maximum voluntary contraction.

Participants received 1-h passive stretching under intelligent control, and pre- and post-evaluations were conducted immediately before and after the intervention. Figure 1 shows the overall flow of the experimental procedure conducted during the study.

### Evaluations

#### Instrumented Tendon Reflex Test

To quantify the reflex components of spasticity, we performed an instrumented tendon reflex test at 60° of elbow flexion. The elbow joint was restricted to an isometric condition to minimize nonreflex torque contributions of joint stiffness, viscosity, and limb inertia, which are dependent on joint motion. Since there was essentially no limb motion, the reflex contribution was readily separated from the minimized non-reflex contributions to joint torque, which otherwise would have been difficult to separate if the limbs could move. A rubber pad (1 cm in diameter) with double-sided adhesive interface was used to perform the tapping of the biceps tendon at a spot where the strongest reflex response was evoked. An instrumented tendon hammer with a force sensor mounted at its head was used to tap the rubber pad. This arrangement transmitted the tendon-tapping force accurately and evenly to the biceps tendon, and the coefficient of variation of the tendon reflex parameters was reduced (34, 35). The participants were seated comfortably during this setup and were asked to completely relax and not react to/anticipate the tapping both before and during the tapping process. The tapping force was increased gradually until a significant muscle contraction was evoked. The biceps tendon was then tapped at approximately that level about ten times during a trial, with random inter-stimulus intervals averaging about 2.5 s. About three trials were conducted. An electromyography (EMG) sensor (Delsys Bagnoli-8, Boston, USA) was mounted on the biceps musculature to measure the muscle response to the instrumented tendon tapping. The tendon tapping force and elbow flexion torque were sampled by a computer at 1 kHz after lowpass filtering (90 Hz cut-off).

#### Passive ROM, Active ROM, and Stiffness

The elbow joint was moved passively in both flexion and extension directions by the stretching device. As the elbow joint was being moved, the angular joint position and passive resistance torque (PRT) generated by the elbow musculature were measured continuously. For the passive ROM (PROM), the stretching device passively moved the elbow in the available range under controlled quantitative peak resistance torques. For evaluation of the stretching-induced improvement, the elbow extension ROM at 10 Nm PRT was used.

For active ROM (AROM), the participants were instructed to actively move their elbow slowly, and the resultant ROM was measured. Three trials of PROM and AROM were conducted and averaged. The PROM measures were divided into flexion ROM and extension ROM. AROM was measured as the total elbow ROM performed by the participant. EMG sensors were mounted on the biceps, triceps, and brachioradialis musculature to measure the muscle actions during AROM.

The elbow joint stiffness at 5° of elbow flexion position during stretching was determined as a non-reflex component. The quantitative data of joint stiffness acquired at the beginning and end of the stretching sessions served as before and after parameters, respectively.

#### Muscle Strength

Muscle strength was also measured with the intelligent stretching device. The participants were instructed to perform isometric maximal voluntary contractions to measure the elbow flexor and extensor strengths, respectively.

## Intervention

### Stretching Under Intelligent Control

**Figure 2:**
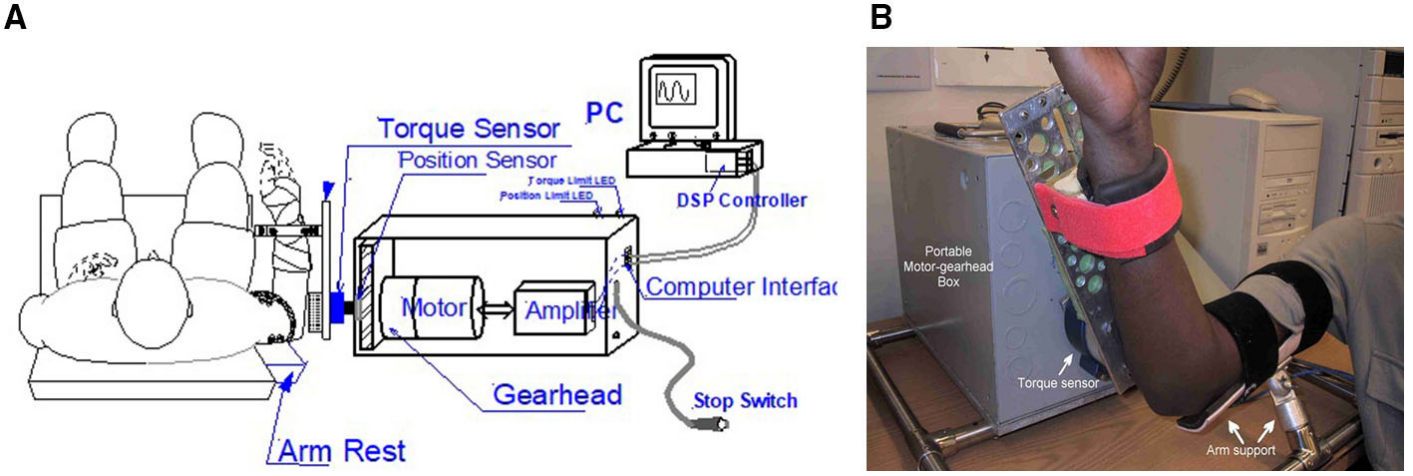
**(A)** The intelligent stretching device designed to stretch the elbow joint with spasticity and evaluate treatment outcome with multiple neuromechanical outcome measures. A digital signal processor (DSP) controller constantly adjusts the stretching velocity based on the resistance, and it checks the joint position and torque signals at 2,000 Hz and shuts down the system if they are out of pre-specified ranges. The torque limit and position limit light-emitted diodes (LEDs) indicate whether a resistance torque limit or position limit (safety limits) is reached, respectively, during the stretching. **(B)** Shows the position of the subject during the stretching.

The intelligent stretching device (Figure 2) was driven by a servomotor controlled by a digital signal processor (DSP), with the Personal Computer (PC) and DSP collecting data and controlling the stretching, respectively (34, 36). During the stretching, the DSP controller read the joint position and resistance torque and controlled the stretching velocity to be inversely proportional to the resistance torque. As the resistance torque increased near the extreme ROMs, the stretching device slowed down, thereby resulting in a slow and gradual stretching of the involved muscle–tendon complex and producing a larger elbow ROM. The stretching device stretched the relatively slack muscles at higher speeds in the mid ranges where the resistance was typically low. However, if the device detected high resistance in the mid ranges, the stretching was accordingly slowed down. Once the specified peak resistance torque or position limit was reached, the stretching device held the elbow joint at that extreme position for a period (~5 s). To ensure safety, the DSP controller constantly checked the joint position and torque signals at 2,000 times per second and shut the system down if they were out of pre-specified ranges. Two mechanical stops were incorporated to restrict and prevent the motor from moving in a range that would over-stretch the spastic joint.

**Figure 3:**
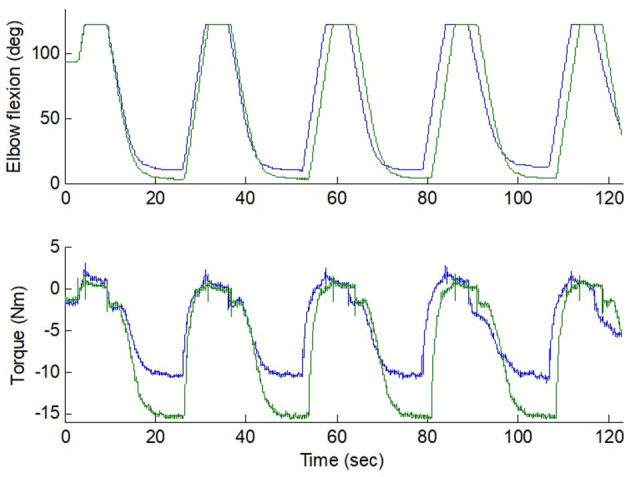
Flexion and torque during two stretching trials (10 Nm and 15 Nm peak torque) in an elbow with strong spasticity. The stretching velocity was reduced gradually down to 0 as the resistance increased. Position limit was reached at the extreme flexion. The blue and green lines represent the flexion angle and torque changes at 10 Nm and 15 Nm peak torque.

During the experiment, the intelligent stretching device performed the stretching in trials, with each trial lasting for about 2 min and stretching parameters adjusted according to the discomfort level of the participants between trials. About 20 trials were conducted for each participant during a session, accounting for a total of 40 min of stretching. During the stretching, the participants were asked to relax and not to react to the stretch (if they did react, the device would reverse its rotation before reaching the extreme positions). A peak velocity (up to 45° per second, only possible at mid-ROM due to the control strategy), peak resistance torque (typical value: 10 N·m), and length of the holding period (typical value: 5 s) at the joint extreme positions were specified and if needed, were conveniently adjusted for each trial. Figure 3 shows a representative flexion angle and torque changes during the strenuous stretching performed on an individual participant.

### Data Analysis

The sampled elbow flexion angle θ(t) and joint torque T(t) were low-pass filtered and sampled at 1 kHz. The EMG signals were filtered with 20–450 Hz bandpass filter. To extract the EMG linear envelope, the raw EMG signals were full-wave rectified and low-pass filtered with 10 Hz cut-off. EMG onset was determined when the EMG linear envelope amplitude exceeded three standard deviations from the mean of the background EMG linear envelope. The background EMG was recorded during a quiet phase before the stretching.

### Reflex Excitability Before/After Stretching

**Figure 4:**
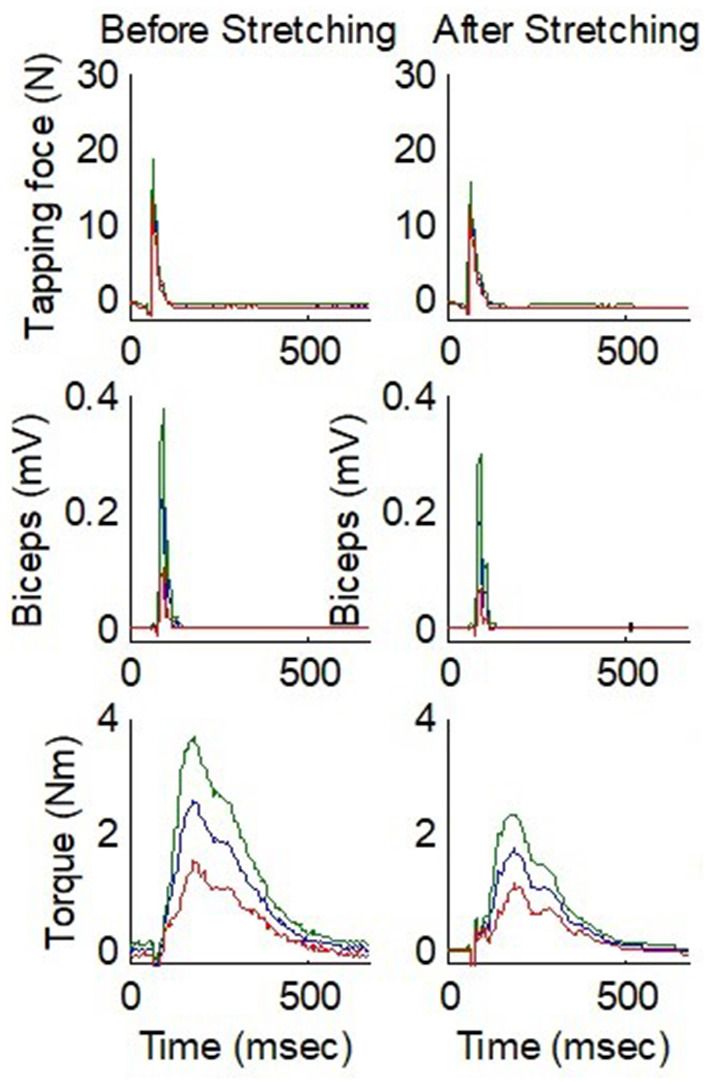
Comparison of before and after stretching for instrumented tendon reflex test results over multiple taps on the biceps tendon of a stroke survivor with spastic elbow. From top to bottom, the three rows show the tendon tapping force, EMG, and elbow flexion torque, respectively. The elbow was fixed at 60° flexion. The blue line represents the mean value and the red and green lines represent the values 1 SD below and above the mean value, respectively. N, Newton; mV, millivolts; Nm, Newton meters; msec, milliseconds.

To quantify the reflex properties, the biceps tendon reflex was viewed in terms of a dynamic input-output relationship. The tendon tapping force was designated as system input and the reflex torque and EMG response were taken as system outputs (9, 37). The impulse response was used to characterize the reflex torque as the output of a system excited by the tendon tapping force. The following physiological parameters were extracted to characterize the impulse response of the tendon reflex system: reflex gain (G_S_), contraction time (t_c_), half-relaxation time (t_hrt_), reflex loop delay (t_d_), contraction rate (R_c_), half-relaxation rate (R_hr_), peak reflex torque (M_p_), and EMG response. Figure 4 shows representative instrumented reflex test results over multiple taps of the biceps tendon with the elbow at 60° flexion.

### Determination of Joint Stiffness

In addition to ROM, joint stiffness was determined as another non-reflex component by following the methods used in previous studies (9, 33, 34, 38).

The anatomic joint angle and torque were plotted to get the torque–angle curves (hysteresis loops). The total number of hysteresis loops ranged from 4 to 8 based on the ROM of the subject. The elbow stiffness at 5° elbow flexion position was assessed as K = ΔT/Δθ, where K is the quasistatic stiffness, and ΔT is the passive torque increment during a certain amount of elbow angular movement (Δθ).

### Statistical Analysis

We performed the Wilcoxon Signed Rank test to compare the before and after stretching effect on passive and active ROM, strength, impulse response parameters of the instrumented reflex test, and non-reflex components. The significance level was set at 0.05. The effect size was calculated using the following formula: *r* = Z/√n, where n indicates the number of observations at two timepoints and Z is the statistic output of Wilcoxon Signed Rank test. The r values of 0.60, 0.40, and 0.20 represent large, medium, and small effect size, respectively (39).

All statistics were performed by SPSS (Version 26; IBM Corp., Armonk, NY, USA).

## Results

### Elbow PROM and AROM

**Figure 5:**
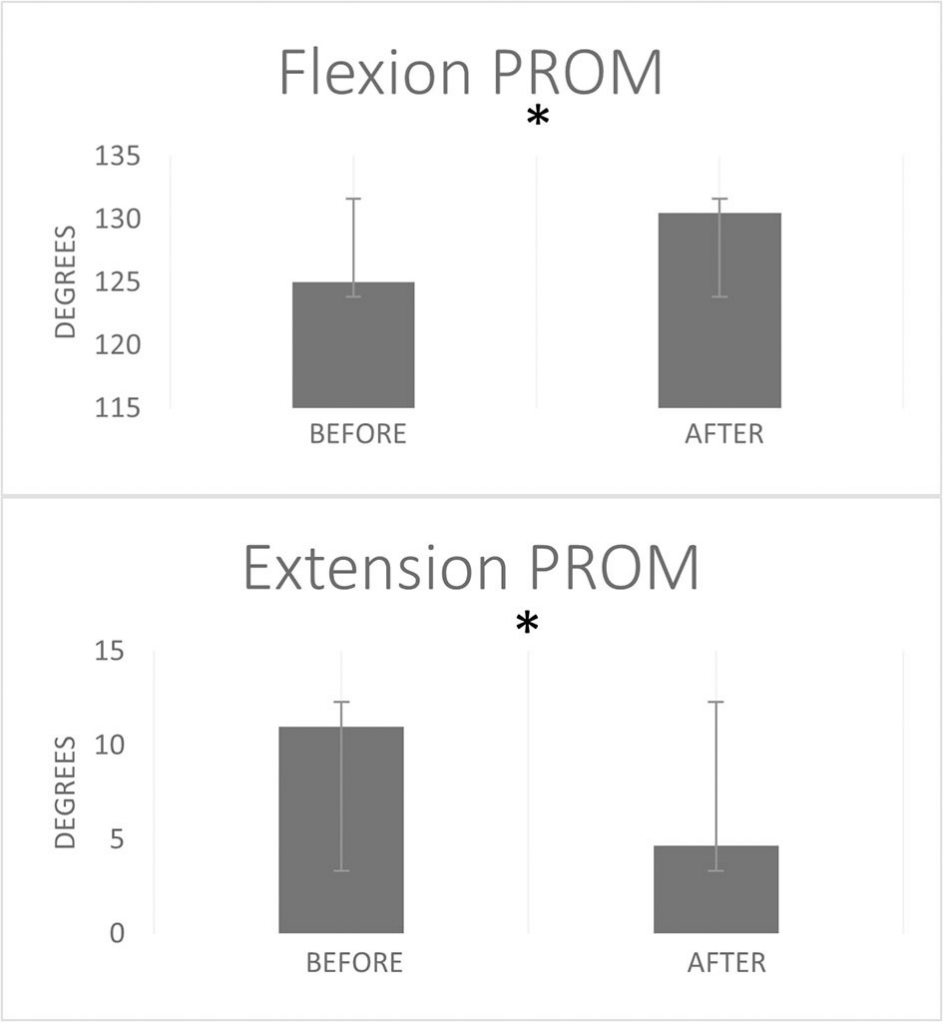
Elbow flexion and extension passive range of motion (PROM) before and after a single stretching session. Error bars represent standard deviation. *Stands for statistically significant difference *p* < 0.05 from Wilcoxon Signed Rank test.

The passive extension ROM showed a significant change after the session of strong stretching; it improved from 11° ± 9.6° (mean ± SD) to 4.6° ± 7.8° (*p* = 0.026, *r* = 0.59). (A full elbow extension corresponds to 0° elbow flexion and the values provided here denote the decrease in this flexion towards 0°). The elbow passive flexion ROM improved from 125.0° ± 4.7° to 130.5° ± 6.3° (*p* = 0.021, *r* = 0.59) (Figure 5). Subjectively, all the eight participants reported having “felt good” about the forceful stretching.

**Figure 6:**
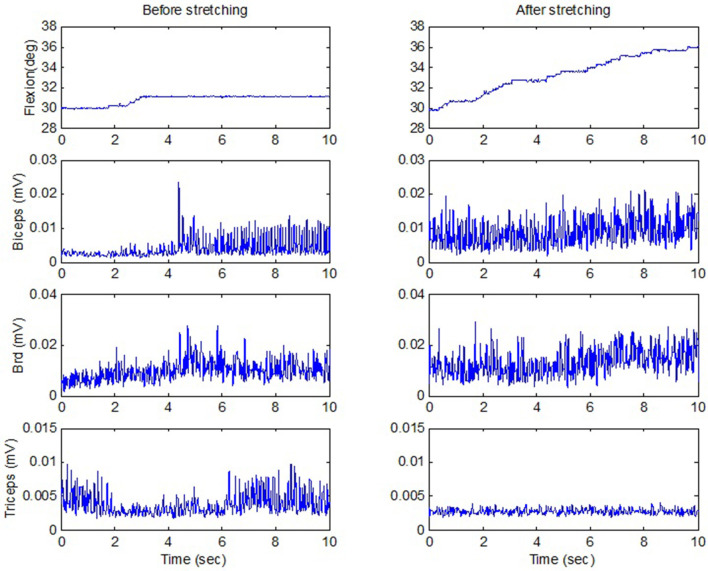
Active ROM when a stroke survivor moved against the passive load of the stretching device including a motor and gearhead (the motor was switched off), before and after stretching. The upward flexion movement was in the vertical plane against gravity. There was considerable reduction of triceps co-contraction (the last row) after stretching, with even stronger biceps contraction. deg, degrees; mV, millivolts; sec, seconds.

The strenuous stretching loosened up the stiff elbows of the participants and helped improve the elbow active ROM. After the forceful stretching, the participants showed a trend of improved active extension ROM from 11.5° ± 10.5° to 7.9° ± 7.8° (*p* = 0.207, *r* = 0.40), and the flexion AROM from 118.4° ± 30.6° to 122.9° ± 32.3° (*p* = 0.391, *r* = 0.25). Functionally, the participants could raise their hands to reach larger ROMs. For example, for an individual participant with spastic elbow and hyperactive reflexes (MAS = 3 for both biceps and triceps, and deep tendon reflex scale = 4), the strenuous stretching loosened the stiff elbow joint and improved voluntary elbow extension from 20° flexion before stretching to 10° flexion after stretching. The stretching increased the AROM partly due to considerably decreased triceps co-contraction during elbow flexion (Figure 6). Functionally, the patients could raise their hands to reach larger ROMs. For the same participant, before stretching, he could move the hand upward in front of the body by 18 cm, which was increased to 28 cm after the intelligent stretching with reduced co-contraction.

### Muscle Strength

The elbow flexion strength showed trends of improvement in the eight participants after the strenuous stretching. Elbow flexor strength showed a trend of increase from 9.62 ± 7.23 Nm to 12.61 ± 7.83 Nm (*p* = 0.129, *r* = 0.43). The extensor strength was 10.03 ± 7.24 Nm before stretching and 10.56 ± 5.19 Nm after stretching (*p* = 0.736, *r* = 0.08). Participants with very stiff joints showed larger increases, while participants with less severe spasticity exhibited lesser increase in strength. The overall strength increase in the eight patients was not statistically significant, given the small sample available. However, similar strenuous stretching of spastic ankles in a larger population of 27 stroke patients resulted in a significant increase in the strength of spastic triceps muscle, which could be related to the loosening up of the accumulated connective tissue and elongated muscle fibers and sarcomeres (40) and a shift in the force–length curves in the spastic muscles to a more optimal operating point (34).

### Tendon Reflex Parameters

Tendon reflex was evaluated immediately before and after stretching using an instrumented tendon hammer and system identification approach (34, 41). The biceps tendon was tapped similarly before and after stretching. Although the stretching did not change any spinal/brain neurological abnormality, reflex-mediated torque was reduced after stretching (Figure 4). However, on comparing the impulse response parameters from the instrumented reflex tendon test performed before and after stretching, we found no significant differences in the reflex-mediated tendon responses. Table 2 gives the impulse response parameters before and after stretching.

**Table 2:** Results from the tendon reflex and passive stiffness analyses.

**Component**	**Parameters**	**Before stretching**	**After stretching**	***p*-value**
Tendon reflex	G_S_ (m·ms)	2.06 ± 1.19	1.8 ± 1.02	0.889
	R_c_ (m/s)	0.40 ± 0.16	0.70 ± 0.38	0.90
	R_hr_ (m/s)	0.35 ± 0.21	0.52 ± 0.32	0.40
	t_c_ (ms)	85.25 ± 20.99	98.75 ± 60.85	0.83
	t_hrt_ (ms)	120.75 ± 56.35	116.50 ± 84.32	0.29
	t_d_ (ms)	24.25 ± 17.78	25.00 ± 17.63	0.94
	M_p_ (Nm)	1.96 ± 1.02	1.88 ± 0.87	0.67
	EMG (mV)	0.28 ± 0.20	0.33 ± 0.22	0.57
Non-reflex	K(Nm/deg)	0.22 ± 0.16	0.16 ± 0.06	0.26

*G_S_, reflex gain; R_c_, contraction rate; R_hr_, half-relaxation rate; t_c_, contraction time; t_hrt_, half-relaxation time; t_d_, reflex loop delay; M_p_, peak reflex torque; K, stiffness at 5° elbow extension angle; m·ms, meter per millisecond; m/s, meter per second; ms, millisecond; Nm, Newton meters; deg, degrees. Values are mean ± SD*.

### Non-reflex Component

The spastic stroke survivors exhibited a trend toward decrement after stretching. However, we did not find statistically significant differences in the decrease of passive stiffness. Table 2 gives the changes in passive stiffness of elbow flexors before and after stretching (K at 5° elbow extension).

## Discussion

The current study focused on the immediate effect of strenuous dynamic stretching performed by the intelligent stretching device on eight chronic stroke survivors with spastic elbow joint. The results indicate that this single session of strenuous dynamic stretching significantly improved the passive elbow ROM with a positive trend toward improvements in the elbow AROM, strength, as well as relieving reflex and non-reflex components of spasticity.

Studies that evaluated the effect of stretching on spastic ankle joints reported positive effects of stretching of ankle plantar flexors in reducing the ankle joint resistance and improving the ankle ROM (33, 34, 36, 40, 42). The results of our study corroborate with the previous studies, and the stretching provided to the elbow flexors could have contributed toward reducing the elbow flexor musculotendinous stiffness, thereby improving both elbow flexion and extension PROMs. Interestingly, few studies have noted that the increase in ROM seen post-stretching could also be a result of increased tolerance to the stretching (43). When reporting the effectiveness of stretching on ROM, most studies have shown improvements usually in PROM (44). The PROM changes seen in our study are consistent with the previous studies.

The improvement in elbow AROM, though not significant, showed good increment post a single session of strenuous dynamic stretching. The improvements could be attributed to reduction in the musculotendinous stiffness. The reduction in passive stiffness with stretching treatment could have loosened the elbow joint, which potentially could have enabled the stroke survivors to move the joint in an increased range (29). Lamontagne et al. (45) found that repeated passive movements had an effect of causing thixotropic changes in the stretched muscles. The dynamic stretching provided in our study could have caused a similar thixotropic change in the elbow musculature leading to improvement in the ROM. Consequently, the dynamic stretching could have elicited activation of the Golgi tendon organ and muscle proprioceptors, thereby causing an improvement in the elbow AROM (46).

Studies by Chung et al., Rydahl et al., and Galvão et al. (9, 47, 48) observed that stroke survivors exhibited higher passive stiffness, which was potentially attributed to connective tissue changes and collagen accumulation leading to fibrosis within the hypertonic muscle. Our stiffness analysis also exhibited a higher passive stiffness in the elbow flexors among all our participants. We were able to quantify the passive stiffness isolated from the reflex properties by moving the elbow joint slowly under precise control. By moving the elbow in this manner, we aimed to minimize the phasic reflex activation. With a single session of strenuous dynamic stretching, we noted a trend toward decrement after the session. The dynamic stretching could have contributed toward decreasing the tightened musculotendinous unit, thereby reducing the muscle stiffness as well as relieving the tension around the surrounding connective tissue and fascia (44).

Although it did not reach a statistical significance, passive stiffness of the spastic elbow flexor showed a trend toward decrement after stretching, which could have resulted from stress relaxation of the spastic muscle under the load applied by the intelligent stretching device. Before stretching, the experimenter set the joint ROM position and torque limits. The stretching could go beyond the position limit by about 5° to allow stretching-induced improvement. The intelligent stretching device stretched the elbow joint until a joint position or torque limit was reached, and maintained the joint position at the limit to induce “stress relaxation,” if the position limit was reached, or “muscle yield” if the torque limit was reached. At the end range of elbow extension, robotic control of the elbow position—usually into further extension—was done at 2,000 times/s based on the joint torque and position. In this experiment, the limits were set to reach a torque limit for a forceful stretching. As a result, the stretching intensity (torque) was kept constant as the joint was pushed into further extension. This was actually “yield”, an increase in joint ROM and muscle length under constant stretching tension. With this characteristic, the intelligent stretching device introduces a genuine way to measure “muscle yield” of human joint *in vivo* condition and provides therapeutic stretching in the most similar way as clinicians do.

Spastic muscles have the tendency to affect the sarcomere length, leading to either their lengthening or shortening as compared to the optimum sarcomere length (49–52). The stretching may have the potential to either increase the length of the spastic muscle or its tendon so that the abnormally lengthened or shortened sarcomeres could possibly regain some of their optimal length and help generate higher force capacity (21, 50, 52–54). The trend toward improvement in both the elbow flexor and extensor muscle strength that we noted could be attributed to this potential change in the sarcomere length caused by stretching. A second potential mechanism for the trend of strength improvements can be attributed to changes in the spinal excitability. It has been argued that one of the mechanisms of spasticity is the increased activity of the Ia afferents. However, several studies suggest II afferent fibers present in the muscle spindle also contribute towards α-motorneurons hyper activation in spastic muscles. The II afferent fibers signals are length-dependent and therefore the stretching could have altered its activation at the resting state and contributed to the increase in isometric strength from resting to activation after of stretching the spastic muscles (7, 55). However, a single session of stretching would not have produced a large and sufficient change in the overall sarcomere length, thereby not showing a significant change in the strength production.

Few studies have quantified the reflex and non-reflex changes in spasticity after stretching. Though our study did not produce statistically significant changes in the impulse response parameters, we did however notice some trend toward change. A recent study comparing the reflex-mediated responses among spastic stroke survivors and healthy individuals showed increased G_S_, R_c_, R_hr_, longer t_c_, and t_hrt_ in stroke survivors (40). The possible mechanisms contributing to these reflex-mediated increments could be the enhanced excitatory synaptic activities of Ia and α-motoneurons along with increased muscle spindle discharge rates (6). Another possible mechanism is the reduced presynaptic inhibition by the descending tracts or inhibition of interneuron inhibitory activity (6, 8). The decrement in G_S_, t_c_, and t_hrt_ after stretching, which is observed in our study, indicates that this single session of intelligent stretching had some effect in decreasing the Ia efferent and α-motoneurons hyperexcitability. The shorter latency period of the t_d_ before stretching could be due to the heightened state of the spastic muscles leading to quicker development of muscle force (35). The increase in this t_d_ latency after a stretching session may further indicate reduction in the hyperexcitability of the muscle. The consequence of these reflex changes in the spastic muscle may present as a decrease in the tonic reflex excitability or an increase in threshold of tonic stretch reflex, subsequently attributing to the muscle-tendon unit length changes with stretching and thus allowing an increase in the elbow ROM (56, 57).

Contrary to our expectation of a decrease in R_c_ and R_hr_, we found that R_c_ and R_hr_ exhibited minute increments in their values post-stretching. This shows that though a single session of stretching could possibly reduce the hyperexcitability, it would require multiple sessions to cause a significant reflex-mediated change.

Several limitations should be acknowledged. First, only eight chronic stroke survivors were included in the pilot study. Studies with a large sample size with high statistical power are needed in the future. Second, in the current study, there is a lack of comparison group which could have provided the reference to interpret the effect of stretching in reducing the hyperactive reflex-mediated responses. Last, we did not examine the duration of retention of stretching effects. Multiple measures with long duration could be utilized for future studies.

In conclusion, a single session of strenuous dynamic stretching significantly improved the passive elbow ROM with a positive trend toward improvements in the elbow AROM, strength, as well as relieving reflex and non-reflex components of spasticity.

## Data Availability Statement

The raw data supporting the conclusions of this article will be made available by the authors, without undue reservation.

## Ethics Statement

The studies involving human participants were reviewed and approved by the Institutional Review Board of Northwestern University. The patients/participants provided their written informed consent to participate in this study.

## Author Contributions

SC and L-QZ designed and conducted the research study. SR, MH, SC, and L-QZ were involved in the analysis of results and prepared the manuscript. All the authors contributed to the article and approved the submitted version.

## Funding

The research was supported in part by the National Institute on Disability, Independent Living, and Rehabilitation Research (90DP099, 90REMM001) and the National Institutes of Health (R42043664, P30AG028747) (USA). MH was supported by a postdoctoral training grant from the National Institute on Disability, Independent Living, and Rehabilitation Research (90AR5028).

## Conflict of Interest

L-QZ holds an equity position in Rehabtek LLC, which received a grant from the National Institutes of Health in developing the intelligent stretching device related to this study. The remaining authors declare that the research was conducted in the absence of any commercial or financial relationships that could be construed as a potential conflict of interest.

## Publisher's Note

All claims expressed in this article are solely those of the authors and do not necessarily represent those of their affiliated organizations, or those of the publisher, the editors and the reviewers. Any product that may be evaluated in this article, or claim that may be made by its manufacturer, is not guaranteed or endorsed by the publisher.
